# Core Decompression With Adinizer-Processed Minimally Manipulated Adipose Tissue for Osteonecrosis of the Femoral Head: A Case Report

**DOI:** 10.7759/cureus.106630

**Published:** 2026-04-08

**Authors:** Yonghyun Yoon, Ji Hyo Hwang, Jaeyoung Lee, Jaewoo Lim, Teinny Suryadi, Anwar Suhaimi, Jonghyeok Lee, Seungbeom Kim, King Hei Stanley Lam

**Affiliations:** 1 Orthopedics, International Academy of Musculoskeletal Medicine, Hong Kong, HKG; 2 Orthopedics, International Academy of Regenerative Medicine, Incheon, KOR; 3 Orthopedics, MSKUS, San Diego, USA; 4 Orthopedic Surgery, Hallym University Kangnam Sacred Heart Hospital, Seoul, KOR; 5 Orthopedic Surgery, Incheon Terminal Orthopedic Surgery Clinic, Incheon, KOR; 6 Orthopedics, Incheon Terminal Orthopedic Surgery Clinic, Incheon, KOR; 7 Physical Medicine and Rehabilitation, Synergy Clinic, Jakarta, IDN; 8 Physical Medicine and Rehabilitation, Hermina Podomoro Hospital, Jakarta, IDN; 9 Rehabilitation Medicine, University Malaya Medical Centre, Kuala Lumpur, MYS; 10 Rehabilitation Medicine, University Malaya, Kuala Lumpur, MYS; 11 Neurosurgery, Bareun Neurosurgery Clinic, Cheongju-si, KOR; 12 Pain Medicine, Miso Pain Clinic, Suwon, KOR; 13 Faculty of Medicine, The Chinese University of Hong Kong, New Territories, HKG; 14 Faculty of Medicine, The University of Hong Kong, Hong Kong, HKG; 15 Board of Clinical Research, The Hong Kong Institute of Musculoskeletal Medicine, Kowloon, HKG

**Keywords:** adinizer, core decompression, hip preservation, microfragmented adipose tissue, minimally manipulated adipose tissue, osteonecrosis of the femoral head

## Abstract

Osteonecrosis of the femoral head (ONFH) is a progressive disorder that may lead to femoral head collapse and eventual total hip arthroplasty, particularly in relatively younger patients. Joint-preserving strategies remain of interest, and biologic augmentation has recently been explored as an adjunct to core decompression.

We report a case of right ONFH treated with core decompression combined with minimally manipulated adipose tissue (microfragmented adipose tissue, MFAT) processed using an Adinizer system. A woman in her 50s presented with a five-month history of right hip pain. Baseline imaging, including coronal and axial MRI sequences, was consistent with Ficat-Arlet stage III ONFH with subchondral collapse. Given the patient’s preference for a joint-preserving approach, core decompression with intraosseous MFAT injection was performed. Adipose tissue was harvested from the lower abdomen, mechanically processed using an Adinizer system, and delivered through the decompression tract into the femoral head.

Clinically, the patient demonstrated improvement over time. The visual analog scale (VAS) pain score decreased from 10 to 1, and the modified Harris Hip Score (mHHS) improved from 61 to 96 during follow-up. No procedure-related complications were reported.

This case suggests that Adinizer-processed MFAT combined with core decompression may be a feasible joint-preserving approach in selected patients with ONFH, even in post-collapse disease. However, given the single-case design, these findings should be interpreted cautiously. Further studies are required to determine reproducibility, optimal indications, and long-term structural outcomes.

## Introduction

Osteonecrosis of the femoral head (ONFH) is a progressive hip disorder caused by impaired blood supply to the femoral head, which may lead to subchondral collapse, secondary osteoarthritis, and eventual need for total hip arthroplasty [1]. ONFH is characterized by compromised blood supply leading to osteocyte death, subchondral bone weakening, and eventual structural collapse of the femoral head. Because many affected patients are relatively young or active, joint-preserving treatment strategies remain an important consideration, particularly before advanced degenerative destruction develops. Treatment is generally stage-dependent, and the 2019 revised ARCO classification is widely used for radiographic and clinical staging of ONFH [2]. Among joint-preserving options, core decompression remains one of the most commonly used surgical procedures, although outcomes may vary depending on the disease stage and the use of biologic augmentation [3]. In particular, its effectiveness in post-collapse (stage III) disease remains limited, as structural compromise of the femoral head may reduce the potential for mechanical and biological recovery.

Recently, biologic augmentation has gained attention as a potential adjunct to core decompression. Various adjunctive strategies have been explored in combination with core decompression, including platelet-rich plasma (PRP), bone marrow aspirate concentrate (BMAC), and bone grafting techniques. This approach is based on the premise that biologic support may help improve the local microenvironment and potentially enhance tissue repair, particularly in cases where structural damage has already occurred. Among these options, adipose-derived products are of particular interest because adipose tissue is relatively accessible and may provide regenerative and immunomodulatory potential. Among minimally manipulated adipose tissue products, microfragmented adipose tissue (MFAT) can be generated at the point of care using mechanical-only processing systems. The Adinizer is a blade-based device that mechanically micronizes lipoaspirate through sequential cutting under minimal pressure without enzymatic digestion and therefore represents one of the mechanically processed MFAT platforms [4,5]. This mechanical processing approach is designed to preserve stromal vascular components and may retain biologically active elements relevant to regenerative applications. In addition to cellular components, such as the stromal vascular fraction, MFAT retains an extracellular matrix scaffold, which may provide structural support and enhance cell retention and local regenerative signaling at the target site. This distinguishes MFAT from isolated cell-based therapies and may have implications for local tissue repair. Although mechanically processed adipose platforms are increasingly being explored in regenerative practice, indication-specific clinical evidence for Adinizer-processed MFAT remains limited, particularly in orthopedic applications. Evidence for MFAT in ONFH is also scarce, and reports describing intraosseous delivery of Adinizer-processed MFAT through a core decompression tract are especially limited. We therefore present a case of right ONFH treated with core decompression combined with Adinizer-processed MFAT, focusing on clinical pain and functional outcomes.

## Case presentation

A woman in her 50s presented to our clinic with right hip pain that had begun five months earlier. Her medical history included asthma and hypertension. She denied any history of corticosteroid use or recent trauma. The patient's height was 149 cm and weighed 49 kg (BMI: 22.1 kg/m²). She reported chronic alcohol consumption approximately five times per week for 30 years and a smoking history of half a pack per day for 30 years. These factors were considered potential risk contributors for nontraumatic osteonecrosis.

**Figure 1:**
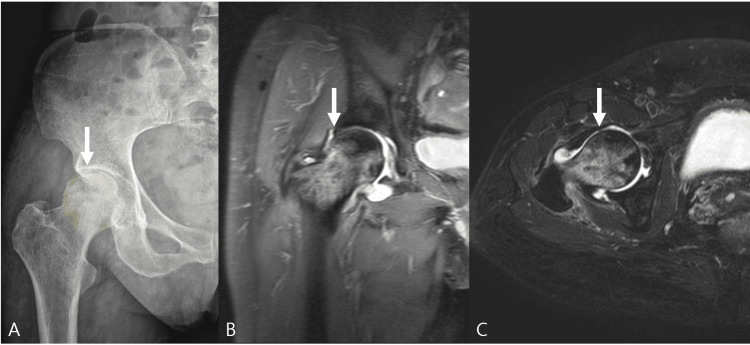
Baseline imaging of the right hip. (A) Anteroposterior pelvis radiograph showing subchondral collapse of the right femoral head (arrow), with the weight-bearing region highlighted. The shaded area indicates the subchondral fracture line (crescent sign), representing early structural failure. (B) Coronal magnetic resonance image demonstrating osteonecrosis with subchondral collapse and associated marrow signal changes (arrow). (C) Axial magnetic resonance image illustrating the extent of femoral head involvement and structural deformity (arrow). Arrows indicate areas of subchondral collapse and necrotic involvement. These findings are consistent with post-collapse osteonecrosis (Ficat-Arlet stage III).

Initial plain radiographs suggested osteonecrosis of the right femoral head. Subsequent external MRI, including coronal and axial sequences, confirmed Ficat-Arlet stage III disease, demonstrating subchondral collapse, marrow signal alteration, and diffuse involvement of the femoral head, particularly in the weight-bearing region (Figure 1).

Her baseline visual analog scale (VAS) score was 10, and the modified Harris Hip Score (mHHS) was 61. Given her age and preference for a joint-preserving approach, core decompression with intraosseous MFAT injection was planned.

Quantitative assessment, such as the Kerboul angle, could not be performed due to the absence of complete orthogonal imaging planes. Nevertheless, the imaging findings indicated extensive subchondral collapse and diffuse femoral head involvement, consistent with advanced (Ficat-Arlet stage III) disease. Other potential causes of hip pain were considered clinically and excluded based on imaging findings and clinical course.

Under sterile conditions, tumescent solution was infiltrated into both lower abdominal regions (60 mL per side; total, 120 mL) for adipose tissue harvesting under local anesthesia, while the hip procedure was performed under spinal anesthesia. A total of 30 mL of purified fat was harvested and mechanically processed using an Adinizer, a blade-based system that sequentially micronizes lipoaspirate without enzymatic digestion, yielding 10 mL of final MFAT. Core decompression was then performed, and the prepared MFAT was delivered intraosseously through the decompression tract into the femoral head. Care was taken to ensure delivery into the necrotic region corresponding to the preoperative imaging findings. The tract was sealed with bone wax at the end of the procedure.

Postoperatively, the patient was discharged on the following day with protected weight bearing, full hip range of motion as tolerated, and ambulation with crutches. Oral anticoagulation therapy was prescribed for three weeks. After six weeks, gradual progressive weight bearing was initiated, and full weight bearing without crutches was achieved by eight weeks. Postoperative medication and rehabilitation followed our previously reported standard protocol for core decompression.

Because the patient resided overseas after treatment, follow-up was conducted primarily via structured telephone interviews using standardized outcome measures. No adverse events, including infection, neurovascular complications, or other procedure-related complications, were reported.

**Figure 2:**
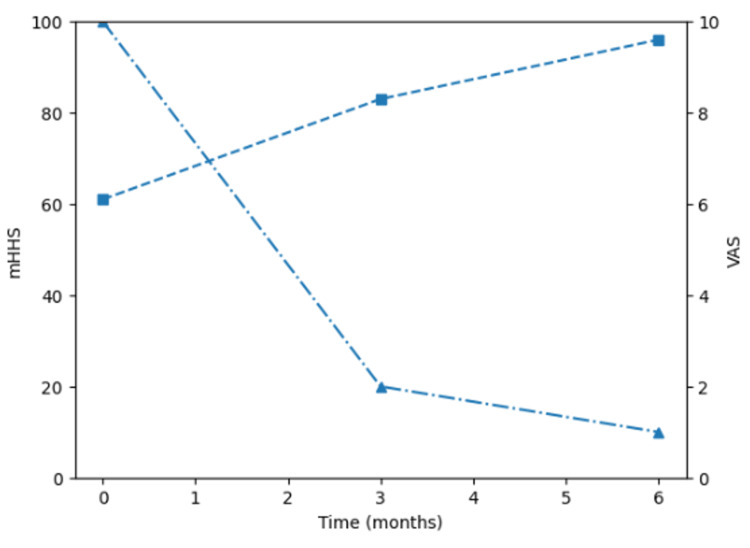
Temporal changes in clinical outcomes. The modified Harris Hip Score (mHHS, left axis) improved from 61 at baseline to 96 at six months, while the visual analog scale (VAS, right axis) decreased from 10 to 1 over the same period.

Clinically, the patient demonstrated gradual improvement over time. Pain decreased from a baseline visual analog scale (VAS) score of 10 to 2 at three months and further to 1 at six months. Hip function was assessed using the mHHS, a widely used functional outcome measure derived from the original Harris Hip Score [6,7]. The mHHS improved from 61 at baseline to 83 at three months and 96 at six months (Figure 2).

According to follow-up communications, these clinical improvements were maintained at 18 and 24 months. However, given that follow-up assessments were performed remotely, objective physical examination and imaging-based evaluations were limited. Therefore, the reported outcomes should be interpreted primarily as patient-reported measures.

## Discussion

The main clinical significance of this case is the intraosseous delivery of Adinizer-processed MFAT as a biologic adjunct to core decompression in post-collapse ONFH. Clinically meaningful symptomatic and functional improvement was observed despite the structurally advanced stage of disease; however, causality cannot be established in a single-case report. The rationale for combining these approaches is biologically plausible. Core decompression may reduce intraosseous pressure and improve local blood flow. However, these mechanisms remain largely theoretical in the context of ONFH and require further validation in controlled clinical studies. Most prior orthobiologic studies in ONFH have focused on bone marrow-derived augmentation rather than adipose-derived products. Randomized and comparative studies have suggested that bone marrow mononuclear cells and cultured bone marrow-derived mesenchymal stem cells used in conjunction with core decompression may improve clinical outcomes or delay radiographic progression compared with core decompression alone [8-10]. Although adipose-derived biologic products have been investigated in various orthopedic conditions, standardized processing methods, delivery techniques, and high-level clinical evidence in ONFH remain limited. Furthermore, most available studies have focused on early-stage disease, and evidence in post-collapse ONFH remains particularly scarce.

Compared with the relatively broader literature on marrow-derived augmentation such as BMAC, adipose-based evidence in ONFH remains limited and heterogeneous. Hong et al. reported that adipose-derived stromal vascular fraction (SVF) injection combined with core decompression and artificial bone grafting was safe and may delay progression in early-stage ONFH, although disease progression still occurred in a substantial proportion of hips [11]. In a separate long-term series, Tantuway et al. described favorable clinical and functional outcomes after intra-osseous autologous SVF implantation with six-year follow-up [12]. More recently, Deng et al. presented a technical note describing Lipogems-based MFAT combined with core decompression for early-stage ONFH, highlighting growing interest in adipose-derived strategies but also underscoring the relative lack of outcome-based data for this approach [13].

An important feature of this case is that treatment was performed in a post-collapse lesion (Ficat-Arlet stage III) rather than in a purely pre-collapse stage. In such cases, structural restoration is unlikely, and the primary goal shifts toward symptom relief and functional preservation rather than radiographic reversal. Therefore, the purpose of treatment was not to demonstrate radiographic reversal, but rather to determine whether clinically meaningful pain relief and functional improvement could be achieved while attempting to preserve the native hip. In this patient, pain improved after treatment, with the VAS score decreasing from 10 to 1, while hip function improved as reflected by an increase in the mHHS from 61 to 96. These findings suggest that patient-centered clinical outcomes may improve in selected cases, even when the structural prognosis remains guarded. In this context, the present case expands the emerging adipose-based literature by describing intraosseous delivery of Adinizer-processed MFAT in structurally advanced ONFH, a setting in which biologic joint-preserving strategies are less commonly reported and clinical expectations are generally guarded.

This report has several limitations. First, it describes a single case and therefore cannot establish causality or generalizability. Second, follow-up was performed largely by telephone because the patient resided overseas, which limited direct physical examination and in-person functional assessment. Third, follow-up radiographs and magnetic resonance imaging were not included in this report. Accordingly, the present case should be interpreted primarily as a clinical and technical feasibility report rather than as evidence of structural regeneration or radiographic disease modification. Despite these limitations, the sustained symptomatic improvement and the absence of reported procedure-related complications support further investigation of this approach in carefully selected patients with ONFH. Future studies incorporating standardized imaging follow-up, quantitative lesion assessment, and controlled comparative designs will be necessary to clarify the role of this technique.

## Conclusions

Adinizer-processed MFAT combined with core decompression may represent a feasible joint-preserving option in selected patients with ONFH. In this case, the procedure was associated with clinical improvement and no reported complications. Further studies are needed to determine reproducibility, optimal indications, and long-term structural outcomes.
